# Anxiety as a Risk Factor for Postoperative Delirium in Elective Spine Deformity Surgeries: A National Database Study

**DOI:** 10.7759/cureus.28984

**Published:** 2022-09-09

**Authors:** Zachary Freedman, Nicholas Hudock, David R Hallan, John Kelleher

**Affiliations:** 1 Neurosurgery, Penn State Health Milton S. Hershey Medical Center, Hershey, USA; 2 Neurological Surgery, Penn State Health Milton S. Hershey Medical Center, Hershey, USA

**Keywords:** elective surgical procedures, post-operative care, delirium, spine surgery, anxiety

## Abstract

Introduction

Generalized anxiety disorder has become one of the most prevalent mental health disorders in the United States. In addition, postoperative delirium has been shown to increase hospital stay, increase mortality, and increased healthcare costs. Few studies have looked at the prevalence of postoperative delirium in patients diagnosed with anxiety undergoing elective spinal deformity procedures. The purpose of this study was to determine if anxiety is a risk factor for postoperative delirium in elective spinal deformity surgeries.

Methods

The authors performed a retrospective analysis using the TriNetX Research Database. Patients diagnosed with kyphosis or lordosis who then underwent elective spinal correction surgeries were identified. This group was then separated based on the diagnosis of a generalized anxiety disorder before the operation versus no diagnosis. Propensity score adjustment, based on mental disorders and other risk factors, was then used to match cohorts on baseline demographics and characteristics. Analysis was performed on the primary outcome of postoperative delirium, with secondary outcomes of upper respiratory tract infections, surgical site infections, sepsis, ventilator dependence, convulsions, stroke, emergency department visits, myocardial infarction, pulmonary embolism, and urinary retention within 30 days after surgery.

Results

Our search included 1,211 patients with a diagnosis of anxiety and 8,055 patients without anxiety. After propensity score matching, 996 patients remained in each cohort. Statistical analysis showed significant outcomes between the matched cohorts in the anxiety group for postoperative delirium (OR 2.788; 1.587-4.899) and convulsions (OR 1.615; 1.006-2.592). All other outcomes were not significant after propensity score matching.

Conclusion

These results showed generalized anxiety disorder is a risk factor for postoperative delirium and convulsions after elective spine surgery. Further research is necessary on the effects of mental health disorders on postoperative delirium and other outcomes to better understand the risks in this population.

## Introduction

The global current prevalence of anxiety disorders is 7.3% and ranged from 5.3% in African cultures to 10.4% in Euro/Anglo cultures [1]. According to United States epidemiological surveys, one-third of the population will be affected by an anxiety disorder during their lifetime, and general anxiety disorder (GAD) has a lifetime prevalence of 6.2% [2]. These anxiety disorders are associated with increased comorbidity and mortality. Patients with spinal deformity suffer a double-edged sword. Both spinal deformity and corrective surgeries have been associated with an increased risk of anxiety [3,4]. The spinal deformity can cause body image dissatisfaction, physical disability, and chronic pain, all of which may limit psychosocial functioning and increase the risk of mental illness [5]. Patients with psychological disorders, such as anxiety disorders, have been identified as a unique population with respect to their higher rates of spinal pain complaints, postoperative complications, and worsened functional outcomes [6].

Postoperative delirium is a common but serious complication of major surgeries. It has been shown to increase hospital stay by 2-3 days, is associated with a 30-day mortality of 7%-10%, and overall increases the cost of healthcare [7]. Due to the quality of care and economic implications of postoperative delirium, previous studies have attempted to determine independent associations and risk factors for the development of postoperative delirium [5]. A recent study identified critical risk factors for the development of postoperative delirium, including truncal surgeries, complex surgeries, emergency surgeries, and surgery on geriatric patients [7]. Despite these known risk factors, the relationship between anxiety disorders and postoperative delirium in spine deformity corrective surgery remains unknown.

This study aims to determine if an anxiety disorder diagnosis is an independent risk factor for the development of postoperative delirium in spine deformity patients following corrective surgery.

## Materials and methods

Study design

This study is a retrospective cohort study database that utilizes the TriNetX Network database to query for patients diagnosed with kyphosis, lordosis, and scoliosis and who underwent spine deformity surgery identified through the current International Classification of Disease-10 (ICD-10) and current procedural terminology codes (CPT). TriNetX is a multi-institutional, de-identified research population cohort derived from electronic medical records that permits query-based data extraction from both inpatient and outpatient encounters. No Institutional Review Board (IRB) approval or patient consent was needed since this study used a de-identified database. Penn State has an agreement in place to access the TriNetX Research Network, and there was no funding associated with this study.

Patient selection

Patients within the TriNetX database were queried for a diagnosis of kyphosis, lordosis (ICD-10 M40), or scoliosis (ICD-10 M41), and the diagnoses of anxiety disorders (cohort 1) (ICD-10 F41) or without the diagnoses of anxiety disorders (cohort 2) with both groups undergoing elective spinal surgeries for their spinal deformities (CPT 1004109). The resulting patients were then reviewed, and propensity scores matched on age, sex, race, ethnicity, alcohol use disorders, nicotine use disorders, depressive disorders, and other mental disorders, including schizophrenia, mood disorders, personality disorders, eating disorders, and impulse disorders. 

Statistical analysis

The primary objective of this study is to identify the risk of postoperative delirium in patients diagnosed with anxiety disorders after elective spinal surgeries. Secondary outcomes include the risk of upper respiratory tract infections (URT), surgical site infections (SSI), sepsis, ventilator dependence, convulsions, stroke, emergency department (ED) visits, myocardial infarction (MI), pulmonary embolism (PE)/deep venous thrombosis (DVT), and urinary retention within 30 days after the surgery. The data was analyzed initially through TriNetX software, which utilizes JAVA, R, and Python programming languages. Additional data analysis and graph formation was performed using Python 3.0 and R-Studio. 

## Results

A total of 1,211 patients were identified in the anxiety cohort, while 8,055 patients were identified in the cohort without anxiety. After propensity score matching, 996 patients remained in each group. At the time of surgery, the mean age for the anxiety group was 30.01 years, while the cohort without anxiety was 30.32 years. Most patients in both groups were female, with 67.97% for the anxiety cohort and 65.06% for the non-anxiety cohort, respectively. Most patients were white at 85.44% and 86.85%. Full demographics and comorbidities are summarized in Table 1.

**Table 1:** Demographics and Comorbidities of Cohorts

Variable	Unmatched					Matched				
Characteristic Name	Anxiety	% of Cohort	No anxiety	% of Cohort	P Value	Anxiety	% of Cohort	No anxiety	% of Cohort	P Value
Number of Subjects	1211		8055			996		996		
Age at Index	33.44		21.12		<0.001	30.01		30.32		0.757
Male	354	29.23%	2709	33.63%	0.002	319	32.03%	348	34.94%	0.169
Female	856	70.69%	5346	66.37%	0.003	677	67.97%	648	65.06%	0.169
White	1048	86.54%	5942	73.77%	<0.001	851	85.44%	865	86.85%	0.364
Black or African American	85	7.02%	960	11.92%	<0.001	74	7.43%	71	7.13%	0.796
Asian	10	0.83%	140	1.74%	0.019	10	1.00%	10	1.00%	1
Native Hawaiian or Other Pacific Islander	0	0%	10	0.12%	0.22	0	0%	0	0%	
American Indian or Alaska Native	10	0.83%	20	0.25%	0.001	10	1.00%	10	1.00%	1
Unknown Race	65	5.37%	988	12.27%	<0.001	59	5.92%	50	5.02%	0.375
Not Hispanic or Latino	1063	87.78%	6453	80.11%	<0.001	879	88.25%	886	88.96%	0.622
Hispanic or Latino	41	3.39%	504	6.26%	<0.001	38	3.82%	41	4.12%	0.731
Unknown Ethnicity	107	8.84%	1098	13.63%	<0.001	79	7.93%	69	6.93%	0.393
Comorbidities										
Hypertensive Diseases	346	28.57%	644	8.00%	<0.001	226	22.69%	183	18.37%	0.017
Heart Failure	54	4.46%	103	1.28%	<0.001	35	3.51%	18	1.81%	0.018
Ischemic Heart Disease	102	8.42%	128	1.59%	<0.001	60	6.02%	32	3.21%	0.003
Atrial Fibrillation	36	2.97%	75	0.93%	<0.001	19	1.91%	19	1.91%	1
Myocardial Infarction	17	1.40%	24	0.30%	<0.001	10	1.00%	10	1.00%	1
Venous Embolism	48	3.96%	75	0.93%	<0.001	35	3.51%	14	1.41%	0.002
Peripheral Vascular Diseases	52	4.29%	73	0.91%	<0.001	28	2.81%	18	1.81%	0.136
Pulmonary Embolism	38	3.14%	24	0.30%	<0.001	20	2.01%	10	1.00%	0.066
Diabetes Mellitus	109	9.00%	194	2.41%	<0.001	63	6.33%	51	5.12%	0.247
Alcohol Dependence	44	3.63%	41	0.51%	<0.001	18	1.81%	17	1.71%	0.865
Nicotine Dependence	161	13.30%	162	2.01%	<0.001	78	7.83%	75	7.53%	0.801
Opioid Dependence	61	5.04%	28	0.35%	<0.001	32	3.21%	10	1.00%	<0.001
Mental Disorders										
Depressive Episode	397	32.78%	189	2.35%	<0.001	191	19.18%	186	18.68%	0.775
Major Depressive Disorder	104	8.59%	21	0.26%	<0.001	51	5.12%	14	1.41%	<0.001
Sleep Disorders	58	4.79%	46	0.57%	<0.001	39	3.92%	10	1.00%	<0.001
Impulse Disorders	35	2.89%	55	0.68%	<0.001	24	2.41%	26	2.61%	0.775
Persistent Mood Disorders	82	6.77%	19	0.24%	<0.001	19	1.91%	18	1.81%	0.868
Eating disorders	27	2.23%	50	0.62%	<0.001	17	1.71%	23	2.31%	0.338
Personality Disorders	24	1.98%	10	0.12%	<0.001	10	1.00%	10	1.00%	1

Outcomes were measured within one month following surgery. After propensity score matching, delirium was significantly increased in the anxiety cohort (2.788 OR; 95% CI, 1.587, 4.899; P<0.001). Additionally, we found the occurrence of convulsions was also significant in this cohort (1.615 OR; 95% CI, (1.006, 2.592); P<0.001). Non-significant outcomes between the groups included URT, SSI, sepsis, ventilator dependence, stroke, ED Visit, MI, PE/VTE, and urinary retention. Full outcomes can be found in Table 2.

**Table 2:** Outcomes Following Elective Spinal Surgery

	Unmatched			Matched		
Variable	Odds Ratio	95% Cl	P Value	Odds Ratio	95% Cl	P Value
Postoperative Complications						
Delirium	7.075	(4.956, 10.101)	<0.001	2.788	(1.587, 4.899)	<0.001
Upper Respiratory Tract Infection	2.289	(1.216, 4.310)	0.008	1.202	(0.517, 2.796)	0.668
Surgical Site Infection	2.062	(1.468, 2.895)	<0.001	1.541	(0.904, 2.627)	0.110
Sepsis	3.086	(2.009, 4.739)	<0.001	1.437	(0.722, 2.862)	0.299
Ventilator Dependence	2.741	(1.832, 4.102)	<0.001	1.284	(0.689, 2.395)	0.430
Convulsions	1.510	(1.114, 2.046)	0.008	1.615	(1.006, 2.592)	0.045
Urine Retention	1.773	(1.385, 2.268)	<0.001	1.379	(0.950, 2.003)	0.090
Postoperative Outcomes						
Emergency Department Visit	1.682	(1.359, 2.082)	<0.001	1.183	(0.863, 1.623)	0.296
Stroke	4.183	(1.894, 9.240)	<0.001	1.000	(0.414, 2.413)	1.000
Myocardial Infarction	2.476	(1.195, 5.128)	0.012	1.000	(0.414, 2.413)	1.000
Pulmonary Embolism/Deep Venous Thrombosis	3.737	(2.615, 5.340)	<0.001	1.666	(0.906, 3.063)	0.097

## Discussion

In this retrospective study of 1,992 patients undergoing elective spine deformity corrective surgery, anxiety disorder emerged as an independent risk factor for the development of postoperative delirium and convulsions. 

Prior literature has demonstrated a pattern of increased risk for postoperative complications in patients with anxiety disorders. In a retrospective study of 325 patients who underwent total hip arthroplasty, patients who were diagnosed with preoperative anxiety using the hospital anxiety and depression scale - anxiety (HADS-A) were found to be almost twice as likely to experience postoperative delirium [8]. Similarly, when Ren et al. examined risk factors for postoperative delirium among 263 patients undergoing elective orthopedic surgery, those diagnosed with anxiety were found to be significantly more at risk of postoperative delirium [9]. Interestingly, in a retrospective analysis of 167 patients, there was no association between preoperative or new‐onset postoperative depression and anxiety symptoms diagnosed using HADS-A and the incidence of postoperative delirium [10]. This study controlled for those with pre-existing diagnoses of mental disease, and thus Wang et al. proposed episodes of preoperative anxiety may increase perceptions of surgical pain and frailty but not cognitive declines [10]. In a retrospective analysis of 183 patients who underwent temporal lobe surgery for epilepsy, those with anxiety were not found to be at increased risk of postoperative seizure or convulsion reoccurrence but were found to report more auras than matched patients without anxiety [11]. Further, Nogueira et al. found that patients with anxiety disorders were significantly more likely to have medication-resistant epilepsy and higher seizure frequency than counterparts with the same seizure disorder diagnosis [12]. Thus, while the extent to which anxiety influences the development of postoperative delirium has not been clearly elicited, our study adds to the growing body of literature suggesting anxiety is a significant risk factor for the development of postoperative delirium and may hold a connection to postoperative seizures and/or convulsions. 

There is a relative lack of research into the connection between anxiety disorders and the development of postoperative delirium. In a study by Kazmerski et al. examining the relationship between oxidative stressors and the development of postoperative delirium, it was found that pre- and postoperative soluble receptors for advanced glycation end-product (sRAGE) levels were significantly higher in the delirium group compared to the non-delirium subjects. It was also found that preoperative sRAGE levels were significantly increased in patients with anxiety disorders [13]. Kazmerski et al. further elicited a significant relationship between decreased oxidative stress capacity and the development of postoperative delirium; however, there were no significant findings of differences between pre- and post-operative antioxidant activity in patients with and without anxiety disorders [13]. The limited data available suggests there may be a link between the development of postoperative delirium and patients who are suffering from anxiety due to increased advanced glycosylation end products. Further data would be needed to definitively support or reject a biological basis for the accumulation of these glycotoxins influencing the outcomes of patients with anxiety disorders.

Due to the impact of postoperative delirium on surgical outcomes, there have been some studies performed on methods to decrease mental health comorbidities in surgical patients. In an article by Stamenkovic et al., it was concluded that patients with preoperative anxiety could benefit from multimodal analgesia, including non-pharmacological methods such as cognitive therapy and music therapy, and relaxation [14]. In a meta-analysis by Bradt et al., there was found to be a significant reduction in the severity of preoperative anxiety with the use of preoperative music therapy, with one study concluding the benefits to be greater than pharmacologic treatment with midazolam [15]. In an additional meta-analysis of preoperative nursing management of patients with anxiety, it was concluded that different types of nursing interventions (educational and informative interview, motivational interview, empathic interview, and hand massage) acted to reduce preoperative anxiety [16]. One shortcoming of the literature on the treatment of surgical patients with anxiety was the transition to examining surgical outcomes. Our study adds further evidence that anxiety is a risk factor for negative postoperative complications, but further research could be completed to understand if this could be alleviated with presurgical anxiety treatments.

This report is not without limitations. The major limitation is the retrospectively nature to look at patients rather than prospectively throughout their hospital course. Additionally, due to using a database, we are not able to look at patient level data on specific outcomes and are only able to view outcomes as a percentage of the cohort. We are also unable to report on radiology information and the spinal cord levels involved in the operation. The data collected within the database is for billing purposes and not for clinical use; thus, some information is inevitably missing.

## Conclusions

In this study, we examined the impact on generalized anxiety disorder in post-operative complications and outcomes in patients undergoing elective spinal deformity operations. The results of this study suggest that generalized anxiety disorder is a risk factor for postoperative delirium and post-operative convulsions after elective spinal surgery. Further prospective investigation is needed to determine the association of these disorders and post-operative outcomes. In addition, further studies are needed to learn more and make proper recommendations to decrease the risk of delirium in this patient population.
